# NOVEL APPROACHES TO MINIMIZE SOCIAL ISOLATION AND MAXIMIZE PARTICIPATION IN OLDER ADULTS WITH DEMENTIA

**DOI:** 10.1093/geroni/igac059.175

**Published:** 2022-12-20

**Authors:** Juleen Rodakowski, Juleen Rodakowski

## Abstract

Older adults experiencing cognitive decline, including those with Alzheimer’s disease and related dementias, are at-risk for social isolation and participation restrictions, especially given the COVID-19 pandemic. Previously examined research has primarily focused on reducing problematic behaviors and remediating cognition for individuals with cognitive impairments. In this symposium, we will discuss novel intervention approaches and under-studied aspects of lifestyle behaviors that minimize social isolation and maximize participation for older adults with Alzheimer’s disease and related dementias. Information presented in this symposium is intended to inform future intervention research to achieve an optimal quality of life for those with cognitive impairments. The first presentation will describe an analysis of lifestyle behaviors and cognition in an older population. The second presentation will discuss the development of an intergenerational, active music intervention that is delivered virtually to individuals with dementia and their caregivers. The third presentation will describe the effects of an adaptive horseback riding program that is becoming increasingly prevalent and improves the quality of life of persons living with Alzheimer’s disease and related dementia.

